# The 2Ih and ^OXO^G Proximity Consequences on Charge Transfer through *ds*-DNA: Theoretical Studies of Clustered DNA Damage

**DOI:** 10.3390/molecules28052180

**Published:** 2023-02-26

**Authors:** Boleslaw T. Karwowski

**Affiliations:** DNA Damage Laboratory of Food Science Department, Faculty of Pharmacy, Medical University of Lodz, ul. Muszynskiego 1, 90-151 Lodz, Poland; boleslaw.karwowski@umed.lodz.pl

**Keywords:** charge transfer, reactive oxygen species, DNA repair, mutagenesis, DFT, 5-carboxamido-5-formamido-2-iminohydantoin, 7,8-dihydro-8-oxo-2′-deoxyguanosine

## Abstract

Genetic information is continuously exposed to harmful factors, both intra- and extracellular. Their activity can lead to the formation of different types of DNA damage. Clustered lesions (CDL) are problematic for DNA repair systems. In this study, the short ds-oligos with a CDL containing (*R*) or (*S*) 2Ih and ^OXO^G in their structure were chosen as the most frequent in vitro lesions. In the condensed phase, the spatial structure was optimized at the M062x/D95**:M026x/sto-3G level of theory, while the electronic properties were optimized at the M062x/6-31++G** level. The influence of equilibrated and non-equilibrated solvent-solute interactions was then discussed. It was found that the presence of (*R*)2Ih in the ds-oligo structure causes a greater increase in structure sensitivity towards charge adoption than (*S*)2Ih, while ^OXO^G shows high stability. Moreover, the analysis of charge and spin distribution reveals the different effects of 2Ih diastereomers. Additionally, the adiabatic ionization potential was found as follows for (*R*)-2Ih and (*S*)-2Ih in eV: 7.02 and 6.94. This was in good agreement with the AIP of the investigated ds-oligos. It was found that the presence of (*R*)-2Ih has a negative influence on excess electron migration through ds-DNA. Finally, according to the Marcus theory, the charge transfer constant was calculated. The results presented in the article show that both diastereomers of 5-carboxamido-5-formamido-2-iminohydantoin should play a significant role in the CDL recognition process via electron transfer. Moreover, it should be pointed out that even though the cellular level of (*R* and *S*)-2Ih has been obscured, their mutagenic potential should be at the same level as other similar guanine lesions found in different cancer cells.

## 1. Introduction

Each cell, whether eucaryotic or procaryotic, is exposed to varying levels of free radical activity. During evolution, oxygen has become the element of life; however, as a result of its radical nature, it can easily form reactive species (ROS) such as a hydroxyl radical (HO•), singlet oxygen (^1^O_2_), superoxide anion radical(O_2_^•−^), hydroperoxide (H_2_O_2_), and so on. [1]. Physiologically, mitochondria are the main source of ROS; approximately 1% of oxygen intake is converted to O_2_^•−^ [2]. This one percent is the effect of an incomplete four-electron O_2_ reduction to a water molecule. Moreover, one of the most reactive molecules known in chemistry, HO•, is produced in vivo via the Haber-Weiss reaction catalyzed by transition metal ions, e.g., Cu, Fe, Mn, Co, etc. [3]. Additionally, cells like neutrophils and macrophages can produce ROS as a defense molecule against pathogens and inflammation by specific processes. On the other hand, organisms are exposed to external factors such as ionization, radiation, xenobiotics, and drugs that can induce ROS. Photons and particles (Low-, High-Linear Energy Transfer (LET)) not only cause water radiolysis but can lead to the oxidation of other molecules if they possess sufficient energy to eject an electron [4]. All information about the miracle of life has been transferred and coded for future generations in the linear sequence of four nucleosides linked by phosphate diester bonds. DNA and other biomacromolecules in a cell are continuously exposed to harmful factors, which can cause lesions. Within all nucleobases, guanine (Gua) has been found to be the most susceptible to oxidation, with an oxidation potential vs. NHE at the level of 1.29 V [5]. The product of a guanine radical depends on the reaction condition (oxidizing or reducing), leading to 8,7-dihydro-8-oxo-2′-deoxyguanosine (^OXO^dG), 2,6-diamino-4-hydroxy-2′-deoxyforamidopirimidine (^Fapy^dG), and further product formation [6].

**Figure 1:**
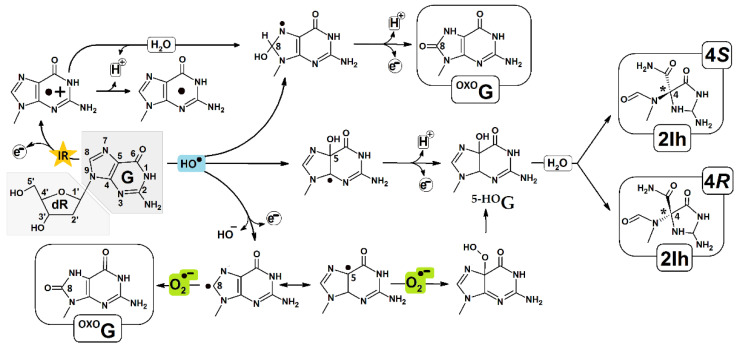
Proposed pathways of 5-carboxamido-5-formamido-2-iminohydantoin (R/S 2Ih) and 7,8-dihydro-8-oxo-2′-deoxyguanosine (^OXO^G) formation.

The oxidizing potential of ^OXO^dG is lower than all canonical nucleosides, i.e., 0.74 V, which makes it more prone to oxidizing by external factors, leading to spiroiminodihydantoin or 5-guanidinohydantoin formation [5]. On the other hand, the interaction of the hydroxyl radical with Gua can lead, after guanine C4 radical rearrangement via an acyl shift, to 5-carboxamido-5-formamido-2-iminohydantoin (2Ih) production. The formation of 2Ih *R* and *S* diastereomers has been observed [7]. The chiral carbon atom in 2Ih corresponds to the C4 of Gua, as shown in Figure 1. The appearance of the above lesions in the genome, if not repaired, has been implicated in pathologic processes, e.g., aging, cancer, neurologic disorders, etc. [8]. Most of the above-mentioned isolated DNA lesions are removed from the genome by base excision repair (BER), which is a highly conservative evolutionary process and is initiated by specific glycosylases. The last one recognizes the lesion in the vast majority of nucleotides [9]. Even though the DNA damage-removing process by mono- or bi-functional glycosylases is well known, the recognition process is still obscure. One theory suggests that these proteins can scan ds-DNA by electron transfer [10]. This mechanism has been investigated and verified for isolated lesions [11]. When clustered DNA damage (CDL) is formed in the genome (as a positive) as a result of radiotherapy or chemotherapy, or when it is induced unintentionally (as a negative) by other harmful external factors, the situation becomes much more complicated. The CDL recognition and repair process are critical for the efficacy of therapy treatment and the safety of genetic information [12]. Therefore, in this article, the influence of clustered lesions containing two different lesions, i.e., (R/S)-2Ih and ^OXO^dG, on charge transfer was taken into consideration.

## 2. Results and Discussion

Numerous factors can induce guanine oxidation in a direct or charge transfer-mediated mode, such as ionization radiation, oxiranes, hydroxyl radicals, Mg or Ni complexes of KHSO_4_, etc. [6]. As shown in Figure 1, the guanine radical (G^●+^) formed is susceptible to hydration or HO●,^1^O_2_ addition [1]. It has been found that C5 carbon of G^●+^ is preferred over C8 for this process, which leads to 5-hydroxy derivative (^5-HO^G) formation. The latter product is unstable in vivo/vitro and, after a 1,2-acyl shift and intermolecular rearrangement, forms 5-carboxamido-5-formamido-2-iminohydantoin (2Ih) as a mixture of the two isomers 4*R* and 4*S* (tnumber four indicates the carbon atom position on the initial guanine). As can be quickly recognized, the structures of ^OXO^G and 2Ih are different because of the reaction paths that lead to their formation [13]. Surprisingly, Fleming et al. have shown that both molecules are formed on par in simulated in vivo conditions, but in the presence of low amounts of a reductant, 2Ih becomes the major product [14]. It should be pointed out here that in normal cells, the mutagenic potential of ^oxo^dG is around 3% if the repair mechanisms are efficient [15]. When the above defense systems fail or are compromised, the situation becomes more dangerous; ^OXO^G can form the pair with dA or dC, resulting in the G:::C→T::A transversion mutation in the second step of genetic material replication. The presence of 2Ih in the template strand leads to opposite dGTP or dATP insertion by a polymerase with a higher promotion for (*R*)2Ih. The above can lead to the G:::C→C:::G or G:::C→A::T transversion. However, the mutagenic potential of both diastereomers has yet to be determined. It can be expected to be at the same level as other guanine lesions due to the fact that similar mutations were found in different cancer cells [16]. Fortunately, 2Ih was found to be the dominant substrate for NEIL1 (“Nei-like” glycosylase in humans) and endonuclease VIII (Neil1 prokaryotic analog) bifunctional glycosylases, which initiated the BER protein cascade. Additionally, it has been discovered that the (*R*)-2Ih diastereomer was removed much more effectively than the (S)2Ih [17]. However, the most intriguing question is how DNA glycosylases effectively discover the DNA lesion in the vast majority of undamaged nucleotides/bases. This question is complicated by the fact that biochemical energy is not necessary for their activity. Several hypotheses for this process have been proposed [18], among which the electron scanning mechanism proposed by Barton looks reasonably promising [19]. Given this, as well as and the fact that DNA damage, lsuch as hydantoins, disrupts the double helix structure, influencing the π-π base stacking interaction, the electronic properties of short oligos containing *R* or *S* diastereomer of 2Ih in combination with ^OXO^G were investigated.

### 2.1. The Influence of Clustered DNA Damage Containing R/S-2Ih and ^OXO^G on the Structure of Short Ds-oligos

**Figure 2:**
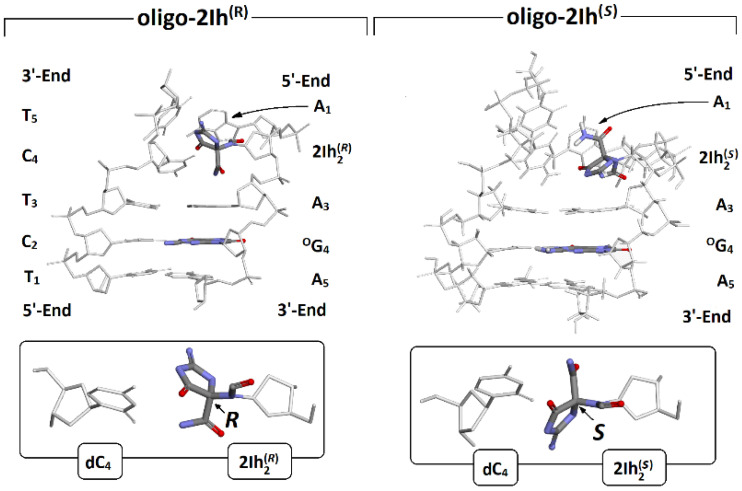
Graphical representation of optimized oligo-2Ih^(R)^, oiligo-2Ih^(S)^ spatial geometries, extracted from double helix base pairs formed by hydantoin and cytidine.

As mentioned in the Materials and Methods section, the geometry of the oligo-2Ih^(*R*)^ and oligo-2Ih^(*S*)^ was optimized to the stationary point using the ONION methodology. For these studies, the modified nucleoside was left at the *anti*-conformation, in which the dihedral angle (O^4^′-C^1^′-N^9^-C^4^) adopted 132.98 and 139.16 [^o^] values for the *S* and *R* diastereomers of hydantoin, respectively. This choice was justified by the fact that initial 2′-deoxyguanosine (which is the precursor of 2Ih) adopted the *anti*-conformation in the canonical double helix structure. For the same reason, ^OXO^G spatial geometry was discussed further in a similar way. As shown in Figure 2, the presence of 2Ih in the ds-oligo leads to significant structural disruption in the place of its formation, irrespective of the diastereomeric forms.

It is particularly visible as differences in local base pair parameters [20]: the buckle and opening of 5′-Enddistal base pair A_1_::T_5_ and the central one A_3_::T_3_, which are located next to (*R*)2Ih_2_::C_4_ and(*S*)2Ih_2_::C_4_ in comparison to canonical G:::C, as shown in Table 1. Furthermore, the presence of hydantoin in the base pair influences hydrogen bonds, shortening the HB-2 and elongating HB-2 by ~0.05 and ~0.1[Å] in comparison to canonical base pairs, respectively Due to the significant structural distortion, no hydrogen bonds were formed and found for the (*R*)2Ih_2_:::C_4_ and (*S*)2Ih_2_:::C_4_ base pairs. However, this effect was propagated over the immediate vicinity and was noted as stronger in the case of an A_3_::T_3_ base pair linked to 3′OH than to the 5′OH (i.e., A_1_::T_5_) group of 5-carboxamido-5-formamido-2-iminohydantoin. Additionally, the structural analysis shows that the presence of an *R* diastereoisomer of 2Ih leads to a more significant double helix structural distortion than that of (*S*)2Ih, which corresponds well with observed Neil1 preferences [18]. Moreover, the *R* diastereoisomer leads to a ds-oligo melting temperature decrease of 16.5, while 4*S* decreases by 16 [°C] in comparison with non-modified ds-oligo [17]. It is noteworthy that the same parameters calculated for the °G_4_:::C_2_ base pair were found at a similar level to those noted for the canonical base pair G:::C. In 1953, Watson, Crick, and Wilkinson discovered the structure of a DNA double helix, which contains two parts: the internal complementary base pairs and an external sugar-phosphate scaffold [21]. It should be noted that base pair π-stacking and hydrogen bonds formed between complementary bases equally stabilized the ds-DNA in the solvation layer. Moreover, the ds-DNA is not a stationary molecule, but rather is in constant motion with charge transfer [22]. The appearance of an excess electron or electron hole leads to an iterative structure fluctuation that should be heightened or preserved by the presence of a DNA lesion. When oligo-2Ih^(*R*)^ and oligo-2Ih^(*S*)^ are compared to their neutral forms after one electron oxidation, the base pair geometry is more sensitive to electron loss in the presence of (*R*)-2Ih than on the opposite diastereomer (Table 1). The following RMSD (Root-Mean-Square Deviation) values were found, 0.31 and 0.25 [Å^2^]. The RMSD values were calculated according to the standard procedure using the BioVia software package [23]. For an outline of the theoretical background, please refer to Arnittali et al. [24]. As expected, the geometry changes after radical cation formation were most visible in the case of the sugar-phosphate backbone, and the RMSD in [Å^2^] adopted the following values: 0.44 for oligo-2IR^(*S*)^ and 0.47 oligo-2IhR^(*R*)^, whereas the extra electron in the ds-DNA structure resulted in radical anion formation. As previously noted, the geometry of the base pair skeleton was found to be the most resistant; the lowest RMSD value of 0.16 [Å^2^] was noted for oligo-2Ih^(*S*)^, the opposite diastereomer; this value was higher by 0.08 [Å^2^] (Table 1). The sugar-phosphate backbone eliminates the constraints raised by the excess electrons settled in the internal part of the double helix. The above has been disclosed by RMSD [Å^2^] values of 0.31 and 0.41 found as results of a comparative analysis between radical anion and neutral forms of oligo-2Ih^(*S*)^ and oligo-2Ih^(*R*)^, respectively. The data presented in Table 1 on the spatial geometry of charged versus neutral oligo forms show that the presence of (*S*)-2Ih makes ds-DNA less susceptible to both electron-hole and extra electron appearances than (*R*)-2Ih. 

### 2.2. The Influence of (R/S)2Ih on the Electronic Properties of the Ds-oligonucleotides Containing Clustered DNA Damage

The initial structure was optimized in the aqueous state, at the M06-2x/D95**:M06-2x/sto-3G level of theory, using the CPCM (conductor-like polarizable continuum model) and ONIOM methodology. The choice of the basis sets is derived from the fact that geometry calculations are less sensitive to the used basis set type than energy/frequencies. Additionally, due to the complex nature of the system, this strategy allowed ground states to be obtained in a reasonable time and at reduced calculation costs. The loss of a valence electron or electron adoption leads to radical formation, which forces changes in the low-unoccupied (LUMO) and high-occupied molecular orbital (HOMO) localizations. Therefore, the ionization potential and electron affinity can be calculated using the orbital energies of the optimized neutral molecules according to Koopman’s theorem [25]. Simply put, the negative values of the HOMO and LUMO energies represent the ionization potential and electron affinity, respectively [26]. Since the charge transfer process passes through a vertical state, the LUMO of the neutral molecule should indicate the in situ location of formation. The subsequent nuclear reorganization gives rise to an adiabatic anion with an adequate HOMO reorganization. The difference between the vertical and adiabatic LUMO localizations can indicate the path of hole transfer within the system [27,28]. Moreover, following Adamoiwiczs’ work, two types of anions can be listed: valence and dipole [29,30]. Since the charge transfer process through the double helix occurs via the π-π interaction between base pairs, vertical or adiabatic anions should show their valence character. The geometries of an accepted molecule should be observed for valence anion types, whereas for dipole bond anion types, an extra electron settles via dipol-dipol interaction, so no effect on the structure is present [29,30]. As previously mentioned for the adiabatic anions, **oligo-2Ih^(*R*)^ and oligo-2Ih^(*S*)^**, some changes in geometries were found and are presented in Table 1. 

#### 2.2.1. Charge and Spin Distribution

In the native ds-oligonucleotides, the positive charge was mainly adopted by purines, while the negative charge was mainly adopted by pyrimidines [31,32,33]. As has been shown previously, the presence of DNA lesions in double helix structures can significantly change the charge distribution, especially when existing in cluster form [34,35]. Here, the short *ds*-oligo with a local multi-damage site containing (*R*) or (*S*) 5-carboxamido-5-formamido-2-iminohydantoin and 7,8-dihydro-8-oxo-guanosine was taken into consideration. The non-equilibrated solvation mode was used to accurately describe the solute-solvent interaction at the initial point of the positive or negative charge appearing within the discussed systems, i.e., oligo-2Ih^(*R*)^ and oligo-2Ih^(*S*)^. For further details, please refer to the valuable work of Sevilla et al. [36]. Moreover, for the charge and spin distribution, the Hirschfeld methodology was applied. One-electron oxidation (electron loss by neutral ds-oligo) of ds-DNA leads to radical cation formation. This process can be induced by different factors, such as ionization radiation, xenobiotics, photosynthetic enzymes, etc. The electron ejection from the double helix initiated the electron-hole, or simple hole, migration process. At the point of its emergence, the gentle equilibrium between solvent and solute became interrupted, and the ds-DNA adopted the vertical cation no-equilibrated (NE) state. As shown in Figure 5, after solvent-solute interaction equilibration (EQ) and geometry rearmament, has been transformed into the vertical cation (equilibrated) and adiabatic cation state, respectively. The last cation after electron adoption is converted to a vertical (NE and EQ) neutral state and finally to an adiabatic one (neutral ground state). This iterative process can be analyzed step by step as the distribution of positive charge and spin within the double helix. The presence of ^OXO^G in the structure of ds-DNA can be perceived as a radical cation sink because its 2′-deoxyguanosine ionization potential is lower than that of other nucleosides/nucleotides. This postulate is commonly accepted in the case of isolated DNA damage considerations [37]. However, nothing is known about clustered lesions containing damage other than ^OXO^G [38]. 

**Figure 3:**
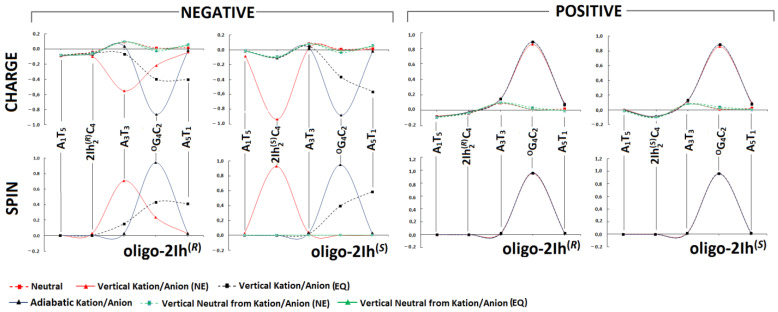
Spin and charge distribution within oligo-2Ih^(*S*)^ and oligo-2Ih^(*R*)^. The *ds*-oligos vertical, or adiabatic, anion and cation forms calculated at the M062x/6-31++G** level of theory in the aqueous phase, by using the non-equilibrated (NE) and equilibrated (EQ) solvent-solute interaction. Only the stacked base pairs were taken into consideration. The raw data is given in Table S1.

As shown in Figure 3, the electron-hole formed in all discussed cases, oligo-2Ih^(*R*)^ and oligo-2Ih^(*S*)^, was located exclusively on the ^O^G_4_:::C_2_ moiety. These results indicate that none of the 5-carboxamido-5-formamido-2-iminohydantoin diastereomers *R* or *S* competes with 7,8-dihydro-8-oxo-2′-deoxyguanosine. The situation became more interesting when the excess electron transfer was taken into consideration. The differences between the influences of the *R* and *S* diastereomers of 2Ih on electron transfer were observed. In the initial step when the vertical anion in a non-equilibrated state was formed, the charge and spin were mainly located at 93% on (*S*)2Ih_2_::C_4_ of oligo-2Ih^(*S*)^, while in the case of oligo-2Ih^(*R*)^, they were dispersed over A_3_::T_3_ and ^O^G_4_:::C_2_ at 71% and 24%, respectively. In the case of oligo-2Ih^(*S*)^, the state of solvent-solute interaction conversion from non-equilibrated to equilibrated causes excess electron dispersion over ^O^G_4_:::C_2_ (39%) and A_5_::T_1_ (59%). The presence of the opposite diastereomer leads to spin dispersion over three base pairs of oligo-2Ih^(R)^: A_3_::T_3_ (15%), ^O^G_4_:::C_2_ (43%), and A_5_::T_1_ (41%). At the final step of the negative charge migration process (adiabatic radical anion), the additional electron almost invariably settled on the ^O^G_4_:::C_2_ base pair independently of the diastereomeric hydantoin form.

#### 2.2.2. Electronic Properties of Oligo-2Ih^(S)^ and Oligo-2Ih^(R)^

The stability of the double helix is the result of three factors: the hydrogen bond between the complementary bases, the stacking between base pairs, and the solvation layer (first hydration shell). It was well visible in the ds-oligonucleotide melting temperature Tm, as described in Breslauer’s invaluable article [39]. On the other hand, these non-covalent interactions are susceptible to structural and electronic ds-DNA changes. The loss of the electron by the molecule leads to its vertical radical cation formation, or if it accepts an extra electron, results in a radical anion. Initially, the solvent-solute disorder (non-equilibrated mode) was observed for this process due to the tight hydration layer. The comparative analysis of the investigated ds-DNA electronic properties revealed that oligo-2Ih^(*S*)^ has a lower VIP^(NE)^ than oligo-2Ih^(*R*)^. However, this difference was negligible (~0.02eV) (see Table 2). Similar results/trends were obtained for VEA^(NE)^. After solvation-solute equilibration, subsequent VIP^(EQ)^ decreases of 0.66 eV were observed in both cases, i.e., oligo-2Ih^(*R*)^and oligo-2Ih^(*S*)^. When VEA^(EQ)^ was taken into consideration, the increases in electron affinity were observed: 0.68eV for oligo-2Ih^(*R*)^and 0.33eV for oligo-2Ih^(*S*)^. The next step of adiabatic anion formation is the charge rearrangement. (The vertical state is converted to adiabatic ones, or as a result of its instability, it is thermalized within 10^−12^ s [40].) A comparison of the calculated adiabatic ionization potential indicates that the AIP of the ds-oligo containing the (*S*)-2Ih moiety is 0.16 eV lower than that found for the opposite diastereomer. The same trend was noted for AEA, but the difference between oligo-2Ih^(*R*)^ and oligo-2Ih^(*S*)^ was found to be smaller, i.e., 0.09eV (Table 2). 

It should be pointed out here that the sugar-phosphate backbone removed from the oligonucleotide structure (as described in the Materials and Methods section) did not change the scheme of the above results. A decrease in the values obtained was observed in each case and was more visible for oligo-2Ih^(*R*)^.

The discovered ‘otherness’ of the discussed oligos raises the question of the electronic properties of single subunits. Due to the base pairs’ isolation/separation from the double helix, the solvent-solute non-equilibrated interaction was omitted, as described above (Table 2). The lowest vertical and adiabatic potentials were observed for the ^O^G_4_::C_2_ moiety; unsurprisingly, for both oligos, these values were almost the same, i.e., 5.93 and 5.53 eV for the VIP and AIP, respectively. For the same base pairs, the highest VEA and AEA were noted in [eV] as follows: −1.49 and −1.93, irrespective of the oligo taken into account. It is important to mention here that the other analyzed base pairs had only negligible differences between vertical and adiabatic ionization potentials as well as electron affinity. Moreover, the above indirectly indicates the thermal hopping mechanism of charge transfer. Conversely, the (*R*)2Ih::C_4_ and (*S*)2Ih::C_4_ showed the highest VEA and AIP values within all the analyzed base pairs as follows: 7.03 and 6.94 eV, respectively. Moreover, the lowest VEA and AEA values, i.e., −1.40 and −1.30 eV, were noted for ^®^2Ih::C_4_ and (*S*)2Ih::C_4_, respectively. All the above indicates that even 7,8-dihydro-8-oxo-2′-deoxyguanosine has a lower ionization potential and a higher electron affinity for the (*R*) and (*S*). The electronic properties of the short ds-DNA fragment as the pentamer are determined by 5-carboxamido-5-formamido-2-iminohydanto. 

### 2.3. The Influence of (R)-2Ih and (S)-2Ih on Charge Migration through Ds-DNA Containing Clustered DNA Damage

**Figure 4:**
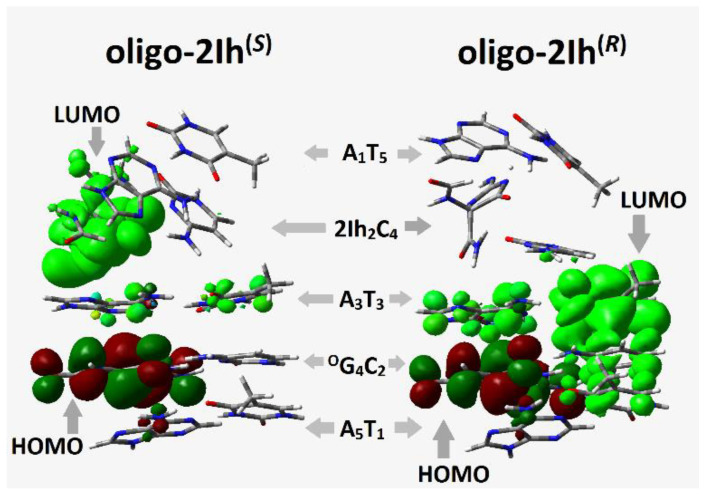
Graphical representation of the molecular orbital distributions (HOMO, LUMO) in neutral states of oligo-2Ih^(*R*)^ and oligo-2Ih^(*S*)^ calculated at the M062x/6-31++G** level of theory in the aqueous phase.

The charge within the double helix passes through π-π stacked base pairs, whose mutual arrangement is crucial for this process. The appearance of *R*- or *S*-2Ih in the ds-DNA structure forces a significant structural change, whereas other CDL components, i.e., ^OXO^G, cause only a negligible geometrical fluctuation. The charge migration can occur through a ds-oligo in its cation radical (oxidative) or anion radical (reductive) state and depends on the base pairs’ electronic properties. According to the Kohn-Sham theory, ionization energy corresponds to Δ*E* = *E*^HOMO^ − *E*^HOMO−1^ and electron affinity to Δ*E* = *E*^LUMO^ − *E*^LUMO+1^. The analysis of valence orbital distributions shows that the HOMO of oligo-2I h^(*S*)^ and oligo-2Ih^(*R*)^ is exclusively settled on the ^OXO^G moiety (Figure 4), whereas the LUMO is found mainly on the (*S*)-2Ih_2_C_4_ subunit of oligo-2Ih^(*S*)^ and on the A_3_::T_3_ base pairs of oligo-2Ih^(*R*)^. The above results suggest the different influences of (*R*) and (*S*) 5-carboxamido-5-formamido-2-iminohydantoin on charge transfer.

Until now, three types of charge transfer have been postulated: tunneling, random walking, and polaron-like hopping [41]. The latter can be discussed as an iterative single-step process between guanines separated by 1–3 A::T base pairs recognized as a bridge. No adenine oxidation was observed [42]. This is in good agreement with the results obtained in these studies. As shown in Table 2, no differences in the calculated VIP/ AIP or VEA/AEA values between the A_1_T_5_, A_3_T_3_, and A_5_T_1_ base pairs were observed. 

However, the presence of 5-carboxamido-5-formamido-2-iminohydantoin in the ds-oligo should lead to significant charge transfer changes depending on the 2Ih diastereomeric form. 

As shown in Table 2, in all cases, the ^O^G_4_:::C_2_ base pair adopts lower VIP and AIP values in both ds-oligos. Additionally, HOMO was found on ^O^G_4_:::C_2_ in the neutral forms of oligo-2Ih^(*S*)^ and oligo-2Ih^(*R*)^. This suggests that the *S* and *R* diastereomers of 2Ih should play similar roles in electron-hole migration. 

The situation becomes different when LUMO is taken into consideration. The lower unoccupied molecular orbit was found on the (*S*)-2Ih_2_C_4_ moiety of oligo-2Ih^(S)^, and on the A_3_T_3_ of oligo-2Ih^(R)^ (Figure 4). The above indicates the different influence of 2Ih on charge transfer through the double helix. However, the charge migration from (*S*)-2Ih_2_C_4_ towards the ^OXO^G_4_C_2_ base pair should be observed. The above assumption is supported by the spin distribution presented in Figure 3 as well as by the calculated VEA/AEA values (Table 2). For both discussed oligos, a higher vertical/adiabatic electron affinity was found for the ^OXO^G_4_C_2_ moiety: −1.48 and−1.93 eV, respectively.

The discussed charge transfer process through the double helix can be described in accordance with the development of Marcus’ theory [43]. The influence of clustered DNA lesions on electron or hole migration can be estimated/quantified by the rate constant (*k*_HT_). This factor, as shown by equation 1, depends on the driving force (ΔG), nuclear reorganization (λ), activation (*E*_a_), and electron-coupling (*V*_12_) energies; all the parameters have been well described in several articles [44,45,46,47]. The Generalized Mulliken–Hush (GMH) methodology was used for electron coupling assignation [48]. Meanwhile, the base pair geometries that were changed by electron loss or adoption can influence the reorganized energy (λ) values (calculation). Subsequently, the driving force (ΔG) of the charge transfer process was defined as the energy difference between the initial and final states. All the above parameters calculated for oligo-2Ih^(*R*)^ and oligo-2Ih^(*S*)^ are presented in Table 3. In all the discussed cases, a significant rise in reorganization energy was noted in the cases of base pairs between which a charge transfer process takes place. For the remainder, the λ values were close to zero eV, which indicates that in the above cases, the transfer occurred without nucleus movement. Surprisingly, the negative values of reorganization and activation energy were discovered in some of the cases presented in Table 3. The above discussed parameters were calculated for the base pair structures isolated from the optimized ds-oligo, which can be different than those found in an ideal model. This has been shown previously in AT crosslink studies [45]. All the data discussed below are presented in Table 3.

The comparative analysis of the charge migration process elucidated that the presence of (*S*)-2Ih, as a part of CDL, slows down hole transfer in comparison with (*R*)-2Ih. The following values in [eV] were found for X_2_C_2_→A_3_T_3:_ 1.06 × 10^2^ and 2.13 × 10^13^ s^−1^ for (*S*) and (*R*)-2Ih oligo, respectively. The reverse was noted for its influence on the hopping process over the (*R*)-2Ih_2_:::C_4_ and (*S*)2Ih_2_:::C_4_ base pairs, noted as A_1_T_1_→ A_3_T_3_. The diastereomer *S* of 2Ih accelerated the charge transfer by around two orders of magnitude in comparison to *R*. The same was noted for the hopping process between (*R*/*S*)2Ih_2_:::C_4_ and ^O^G_4_:::C_2_. Moreover different preferences for hole migration were found for oligo-2Ih^(*S*)^ and oligo-2Ih^(*R*)^, i.e.,A_3_T_3_←A_5_T_1_and A_3_T_3_→A_5,_ respectively.

The analysis of excess electron transfer through ds-DNA only showed significant energy activation for the (*S*)2Ih_2_::C_2_→A_3_::T_3_ process (6.84 eV), while for the opposite diastereomer, it was close to zero [eV] (Table 3). This observation indicates that (*S*)-2Ih can affect the negative charge migration, i.e., *k*_HT_ = 0 [s^−1^] of **X**_2_C_4_→A_3_T_3,_ which was found. This can be supported by the charge and spin distribution, which is presented in Figure 3, and the LUMO localization of the oligo-2Ih^(*S*)^ neutral form (Figure 4). The electron hopping process (*S*)2Ih_2_::C_4_→^O^G_4_:::C_2_ with *k*_HT_ = 1.2 × 10^14^ [s^−1^] can compensate for the above (slowing down). The kHT of the related base pairs was three orders of magnitude lower in the case of oligo-2Ih^(*R*)^: 1.9 × 10^11^ [s^−1^]. As previously described, the electron transfer in the ds-oligo part [A^oxo^GA]*[TCT], which contained 8-oxo-G, was not affected by the presence of a second lesion, i.e., (*R*/*S*)2Ih, in the structure of clustered DNA damage.

## 3. Materials and Methods

The starting geometries of short ds-DNAs were built using the BioVia Discovery Studio v20.1.0.19295 software [23]. They were noted as follows: d[A_1_2Ih^(*R*)^_2_A_3_^O^G_4_A_5_]*d[T_5_C_4_T_3_C_2_T_1_] and d[A_1_2Ih^(*S*)^_2_A_3_^O^G_4_A_5_]*d[T_5_C_4_T_3_C_2_T_1_] as oligo-2Ih^(*R*)^ and oligo-2Ih^(*S*)^, respectively. The M06-2X functional was used for all calculations due to its relative cost-effectiveness and efficiency, as outlined in the Supplementary Materials. Furthermore, in the Supplementary Materials, the rationale for this choice of functional was explained.

The negative charges of the phosphate groups were neutralized by the addition of protons, while the other atoms were saturated by additional hydrogen atoms when it was necessary. This applied strategy is well recognized as applicable to proton/charge transfer or structural studies of DNA [49,50]. Additionally, in his studies, Leszczynski adopted the above procedure for low electron migration from the base moiety to the sugar-phosphate skeleton [51]. The structure optimizations of ds-pentamers were performed using the ONIOM (**O**ur own **N**-layered **I**ntegrated Molecular **O**rbital and Molecular **M**echanics) strategy. The structures of *ds*-oligos were divided into high-HL (nucleobases, M06-2X/D95**) and low-LL (sugar-phosphate backbone, M06-2X/sto-3G) levels of calculation [52,53,54]. All calculations were performed in the condensed phase using the Tomasi’s polarized continuum model (PCM) with a water dielectric constant ε = 78.4. All energy calculations were performed in the aqueous phase according to the density functional theory (DFT). The M062x functional was chosen for complete oligonucleotides with an augmented polarized valence double-ζ basis set of 6-31+G(d,p) and 6-31++G** after sugar-phosphate backbone removal [51]. The sugar-phosphate backbone was removed from the obtained structures, leaving suitable base pair systems with subsequent atom saturation with the necessary hydrogen atoms. The above strategy was chosen to keep the base pair leader in the mutual position as in the double helix structure. Otherwise, without a sugar-phosphate backbone, the base pairs adopt a relaxed geometry, different from that observed in a ds-DNA structure affected by ^Fapy^dG. The added hydrogen atoms for saturation were optimized in the aqueous phase at the M06-2X/D95** level of theory (with the remaining atoms frozen). For all the optimized geometries, a charge and spin analysis was achieved using the Hirshfeld methodology at the M06-2X/6-31++G** level of theory in the aqueous phase [55]. The electronic properties of molecules were calculated as described previously; for further details, please see the reference [56]. The characterization of the transition dipole moment of excited states and the single point calculation at the M06-2X/6-31++G** level of theory were performed using time-dependent DFT (TD-DFT) methodology [57]. Electron coupling was calculated using the Generalized Mulliken-Hush methodology [58]. The solvent effect was looked at in two modes following a previously described methodology, i.e., non-equilibrium (NE) and equilibrated (EQ) polarizable continuum model (PCM) [59,60]. The energy of the molecule in non-equilibrated solvent-solute interaction mode was calculated using two-step processes according to the save-read procedures [59].

**Figure 5:**
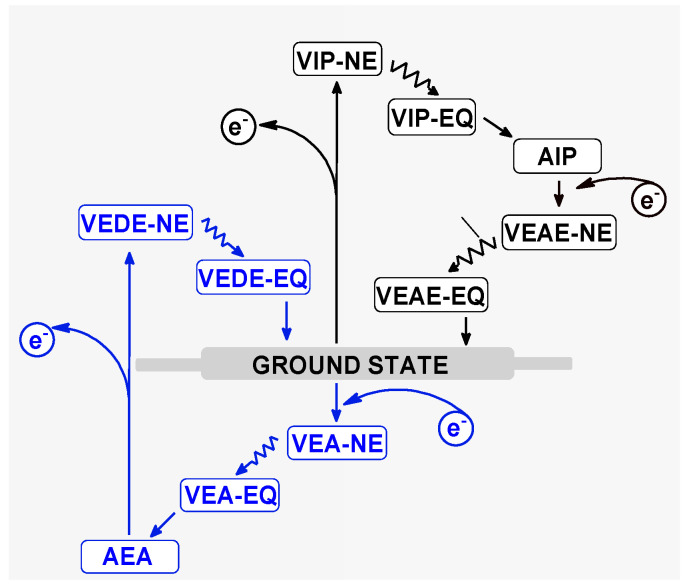
Graphical representation of electronic states and energie [eV] changes during the electron/hole migration through ds-DNA. The abbreviations have been given in the Materials and Methods section.

The following energy notation presented in Figure 5 was used: *E*_geometry_^charge^ of the molecule (neutral form) is described as *E***_0_^0^**, the vertical cation/anion as *E***_0_^+^**/*E***_0_**, the adiabatic cation/anion as *E***_+_^+^**/⁺*E*_−_^−^ and the vertical neutral formed from the cation/anion state as *E**_0_*****^+^**/*E***_0_^−^**. The difference, given in eV, between the mentioned energies corresponds to the suitable electronic states described as follows: **VIP − NE**= *E**_0_*****^+(NE)^** − *E**_0_*****^0^** (vertical ionization potential in the NE state); **VIP − EQ**= *E**_0_*****^+(EQ)^** − *E**_0_*****^0^** (vertical ionization potential in the EQ state); **AIP** = *E**_+_*****^+^** − *E**_0_*****^0^** (adiabatic ionization potential); **VEAE − NE**= *E**_+_*****^+^** − *E**_+_*****^0(NE)^** (vertical electron attachment energy in the NE state); **VEAE − EQ**=*E**_+_*****^+^ –** *E**_+_*****^0(EQ)^** (vertical electron attachment energy in the EQ state); **VEA − NE** = *E**_0_*****^−(NE)^** − *E**_0_*****^0^** (vertical electron affinity in the NE state); **VEA − EQ** = *E**_0_*****^−(EQ)^** − *E**_0_*****^0^** (vertical electron affinity in the EQ state); **AEA** = *E***_0_^0^ –** *E*_−_^−^(adiabatic electron affinity); **VEDE − NE** = *E***_–_^−^ –** *E**_-_*****^0(NE)^** (vertical electron detachment energy in the NE state); and **VEDE − EQ** = *E***_–_^−^** − *E**_-_*****^0(EQ)^** (vertical electron detachment energy in the EQ state). All the above calculations were performed with the Gaussian G16 (version C.01) software package [13]. 

## 4. Conclusions

Genetic information is continuously exposed to harmful factors (intra- and extracellular). Their activity can lead to the formation of various types of DNA damage. Within the vast majority of cases of DNA damage, CDLs are more problematic than isolated lesions for DNA repair systems. It should be pointed out here that CDL numbers in the cells increase during radiotherapy, leading to cancer cells becoming more vulnerable to other factors and therefore resulting in a better medical prognosis for patients. In these studies, the short *ds*-oligos with CDLs containing (*R*) or (*S*)-2Ih and ^OXO^G in their structures were chosen, i.e., oligo-2Ih^(*S*)^ and oligo-2Ih^(*R*)^. The ^Fapy^G and ^OXO^G were found to be the most abundant oxidative guanosine lesions in vitro.

- The ground-state molecule geometries were optimized using ONIOM methodology and the CPCM solvation model (M06-2x/D95**:M06-2x/sto-3G). All requested energies for theoretical studies were performed at the M06-2x/6-31++G** level of theory in the aqueous phase with equilibrated and non-equilibrated solvent-solute interactions.
- The spatial structure comparative analysis between cationic, anionic, and neutral forms showed that the presence of (*R*)-2Ih in the *ds*-oligo structure makes the double helix more sensitive to electron loss or adoption. Additionally (*R*)-2Ih::C forces significant changes in the geometry of the neighboring base pairs (5′-end site of 2Ih) with the standard DNA reference frame parameter. Buckle adopted a value at the level of −32° in comparison to −4° found in oligo-2Ih^(*S*)^.
- Both diastereomers of 5-carboxamido-5-formamido-2-iminohydantoin exerted the same influence on the positive charge and spin distribution within the double helix containing the CDL. The radical cation in all the discussed cases settled exclusively on the ^OXO^G:::C base pairs, as expected. A difference in the influence of (*R*) and (*S*)-2Ih on negative charge distribution was found when the non-equilibrated mode of the vertical anion was taken into consideration, i.e., in the oligo-2Ih^(*S*)^, the negative charge and spin were mainly noted on the (S)-2Ih::C moiety while in the case of oligo-2Ih^(*R*)^, no charge or spin were observed on the 2Ih::C base pair.
- A global electronic properties calculation revealed that oligo-2Ih^(*S*)^ has a lower adiabatic ionization potential (5.53 eV) than oligo-2Ih^(*R*)^ (5.67 eV) and a higher adiabatic electron affinity (2.09 eV) than oligo-2Ih^(*R*)^ (2.0 eV). A careful analysis of the electronic properties of isolated base pairs gave these AIP values for 2Ih^®^::C (7.02 eV) and 2Ih^(*S*)^::C (6.94 eV) base pairs, while the lowest value was assigned for ^OXO^G::C (6.62 eV) in both of the discussed ds-oligos. It should be pointed out that the AEA parameter adopted a higher absolute value in the case of 2Ih^(*R*)^::C (−1.4 eV) than in the case of 2Ih^(*S*)^::C (−1.30 eV).
- The comparative analysis of charge transfer rate, according to Marcus’s theory, elucidated that the presence of (*S*)-2Ih slows down the electron-hole and excess electron migration from the place of formation in comparison with (*R*)2Ih.

Finally, the results presented in the article show that both diastereomers of 5-carboxamido-5-formamido-2-iminohydantoin should play a significant role in the CDL recognition process via electron transfer. Moreover, it should be pointed out that even though the cellular level of (*R* and *S*)-2Ih has been obscured, their mutagenic potential should be predicted on the same level as other guanine lesions found in different cancer cells. Taking all the above into account, it can be postulated that even simple single-stranded clustered DNA damage can significantly change the electronic properties of ds-oligo. Additionally, CDL can influence the charge transfer through the double helix, subsequently disturbing the recognition and repair processes of DNA lesions. Since increases in CDL formation are observed during radio- or chemotherapy, understanding their role in the above processes can be crucial to safe and effective medical cancer treatment.

## Figures and Tables

**Table 1:** The structural local base pair parameters in [°] according to the standard DNA reference frame and hydrogen bond length in [Å]. Hydrogen bond notation: HB-1: Ade(N^1^), Thy(N^3^), Gua(N^6^), and Cyt(O^4^); HB-2: Ade(N^6^), Thy(O^4^), Gua(N^1^), and Cyt(N^3^); HB-3: Gua(N^2^) and Cyt(O^2^). Root-Mean-Square Deviation (RMSD) of atomic positions in [Å^2^], calculated for oligo-2Ih^(*R*)^ and oligo-2Ih^(*S*)^in their Neutral, Anionic, and Cationic forms.

ds-DNA	Base Pair	Structural Parameters					Spatial Geometry Comparison (RMSD Analysis)			
		Buckle	Opening	HB-1	HB-2	HB-3				
oligo-2h^(*R*)^	A_1_::T_5_	−32.84	5.61	3.06	2.80		Form	Overlap	oligo-2Ih^(*S*)^	oligo-2Ih^(*R*)^
	A_3_::T_3_	11.05	7.32	3.10	2.77		Anion versus Neutral	Total	0.25	0.34
	°G_4_:::C_2_	−0.89	−1.92	2.83	2.91	2.89		Base	0.16	0.24
oligo-2h^(*S*)^	A_1_::T_5_	−4.22	0.52	2.95	2.84			Frame	0.31	0.41
	A_3_::T_3_	11.81	4.74	3.05	2.80		Cation versus Neutral	Total	0.36	0.40
	°G_4_:::C_2_	−4.43	−1.45	2.83	2.90	2.87		Base	0.25	0.31
Canonical *ds*-oligo	A::T	5.61	−0.87	2.92	2.85			Frame	0.44	0.47
	G:::C	−0.51	−1.50	2.88	2.88	2.86				

**Table 2:** Electronic properties of oligo-2Ih^(*R*)^ and oligo-2Ih^(*S*)^ as well as isolated base pairs (BP): Vertical (VIP), Adiabatic Ionization Potential (AIP), Vertical (VEA), and Adiabatic (AEA) Electron Affinity are calculated at the M062x/6-31++G** level of theory in the aqueous phase. NE—non-equilibrated solvent-solute interaction; NE—equilibrated solvent-solute interaction. All data are given in [eV].

Base Pair	Oligo-2Ih^(*S*)^ X = (*S*)2Ih				Oligo-2Ih^(*R*)^ X = (*R*)2Ih			
	VIP	AIP	VEA	AEA	VIP	AIP	VEA	AEA
A_1_::T_5_	6.67	6.67	−1.42	−1.42	6.73	6.74	−1.41	−1.40
X_2_::C_4_	6.93	6.94	−1.30	−1.30	7.03	7.02	−1.37	−1.40
A_3_::T_3_	6.65	6.62	−1.39	−1.40	6.79	6.64	−1.38	−1.37
^O^G_4_:::C_2_	5.93	5.53	−1.49	−1.93	5.94	5.54	−1.48	−1.93
A_5_::T_1_	6.66	6.62	−1.43	−1.40	6.65	6.62	−1.44	−1.41
*ds*-oligo	6.65^(NE)^, 5.99^(EQ)^	5.51	−1.07^(NE)^, −1.40^(EQ)^	−2.09	6.70^(NE)^, 6.04^(EQ)^	5.67	−1.00^(NE)^, −1.68^(EQ)^	−2.00
*ds*-BP	6.53^(NE)^, 5.90^(EQ)^	5.50	−0.65^(NE)^, −1.37^(EQ)^	−2.09	6.56^(NE)^, 5.94^(EQ)^	5.57	−0.78^(NE)^, −1.31^(EQ)^	−1.84

**Table 3:** The ΔG, λ, *E*_a_, *V*_12_, and *k*_HT_ of permissible electron-hole and excess electron transfer between base pairs of oligo-2Ih^(*S*)^ and oligo-2Ih^(*R*)^, calculated at the m062x/6-31++G** level of theory in the aqueous phase. Arrows indicate the direction of charge migration.

Excess Electron Transfer											
	Oligo-2Ih^(*S*)^					Oligo-2Ih^(*R*)^					
X = (*S*)2Ih	λ	G	*E*_a_	*V*_12_	*k*HT	X = (*R*)2Ih	λ	G	*E*_a_	*V*_12_	*k*HT
A_1_T_5_←**X**_2_C4	−0.02	−0.12	−0.27	0.08	---	A_1_T_5_←**X**_2_C_4_	0.04	−0.01	0.01	0.04	8.82 × 10^13^
**X**_2_C_4_→A_3_T_3_	0.00	−0.10	6.84	0.04	0.00	**X**_2_C_4_→A_3_T_3_	0.04	−0.03	0.00	2.26	3.97 × 10^17^
A_3_T_3_→OG_4_C_2_	0.44	−0.53	0.00	0.04	3.47 × 10^13^	A_3_T_3_→OG_4_C_2_	0.46	−0.55	0.01	0.05	4.87 × 10^13^
OG_4_C_2_←A_5_T_1_	0.43	−0.53	0.00	0.06	8.24 × 10^13^	OG_4_C_2_←A_5_T_1_	0.43	−0.52	0.00	0.06	7.27 × 10^13^
A_1_T_5_←A_3_T_3_	−0.01	−0.02	−0.02	0.09	---	A_1_T_5_←A_3_T_3_	−0.02	−0.02	−0.02	0.03	---
**X**_2_C_4_→OG_4_C_2_	0.43	−0.63	0.02	0.11	1.2 × 10^14^	**X**_2_C_4_→OG_4_C_2_	0.43	−0.53	0.01	0.003	1.9 × 10^11^
A_3_T_3_→A_5_T_1_	−0.03	−0.002	−0.01	0.07	---	A_3_T_3_→A_5_T_1_	−0.02	−0.04	−0.04	0.07	---
**Electron-Hole Transfer**											
A_1_T_5_←**X**_2_C_4_	0.00	−0.28	33.56	0.19	0.00	A_1_T_5_←**X**_2_C_4_	0.00	−0.28	−5.82	0.10	---
**X**_2_C_4_→ A_3_T_3_	0.03	−0.32	0.81	0.23	1.06 × 10^2^	**X**_2_C_4_→A_3_T_3_	0.16	−0.38	0.08	0.11	2.13 × 10^13^
A_3_T_3_→OG_4_C_2_	0.38	−1.09	0.32	0.35	1.13 × 10^10^	A_3_T_3_→OG_4_C_2_	0.39	−1.10	0.32	0.42	1.82 × 10^10^
OG_4_C_2_← A_5_T_1_	0.37	−1.09	0.35	0.37	4.72 × 10^9^	OG_4_C_2_←A_5_T_1_	0.36	−1.08	0.35	0.35	4.21 × 10^9^
A_1_T_5_→ A_3_T_3_	0.03	−0.05	0.002	0.19	3.1 × 10^15^	A_1_T_5_→A_3_T_3_	0.16	−0.10	0.01	0.03	3.5 × 10^13^
**X**_2_C_4_→OG_4_C_2_	0.39	−1.41	0.66	0.53	4.6 × 10^4^	**X**_2_C_4_→OG_4_C_2_	0.41	−1.48	0.69	0.04	1.0 × 10^2^
A_3_T_3_← A_5_T_1_	0.03	−0.003	0.01	0.01	2.9 × 10^12^	A_3_T_3_→A_5_T_1_	0.02	−0.02	0.00	0.07	5.8 × 10^14^

## Data Availability

Data is contained within the article or Supplementary Materials.

## Supplementary Materials

The following supporting information can be downloaded at: https://www.mdpi.com/article/10.3390/molecules28052180/s1. Table S1: Hirshfeld charge and spin distribution in the shape of *ds*-oligonucleotides, only nucleoside bases were taken into consideration, calculated at the M06-2x/6-31++G** level of theory in the aqueous phase. Vertical Cation (VC^NC^) (NE-non-equilibrated), Vertical Cation (VC^EQ^) (EQ-equilibrated), Vertical Anion (VA^NE^), Vertical Anion (VA^EQ^), Adiabatic Cation (AC), Adiabatic Anion (AA), and Vertical Neutral from Cation (VNC^NE^), Vertical Neutral from Cation (VNC^EQ^), Vertical Neutral from Anion (VNA^NE^), Vertical Neutral from Anion (VNA^EQ^); Table S2: The energy barriers (in eV) for radical cation migration between base pairs within trimers. Vertical (Vert) mode, i.e., the energies of each base pair’s radical cation, which were calculated for their neutral geometry. Adiabatic (Adia) mode, i.e., the energies of each base pair’s radical cation were calculated for their cation geometry. Arrows indicate the direction of Electron-hole or Excess Electron Transfer from one base pair to another, e.g., A^+^→ G calculated at the M06-2x/6-31++G** level of theory in the aqueous phase; Table S3: The energies (in Hartree) of Neural, Vertical Cation, Adiabatic Cation, and Vertical Neutral forms of base pairs extracted from *ds*-oligonucleotides calculated at the M06-2x/6-31++G** level of theory in the aqueous phase; Table S4: The energies (in Hartree) of Neural, Vertical Cation (VC^NC^) (NE-non-equilibrated), Vertical Cation (VC^EQ^) (EQ-equilibrated), Vertical Anion (VA^NE^), Vertical Anion (VA^EQ^), Adiabatic Cation (AC), Adiabatic Anion (AA) and Vertical Neutral from Cation (VNC^NE^), Vertical Neutral from Cation (VNC^EQ^), Vertical Neutral from Anion (VNA^NE^), Vertical Neutral from Anion (VNA^EQ^) of complete DNA double helix and base pairs skeleton extracted from *ds*-oligonucleotides calculated at the M06-2x/6-31+G** and M06-2x/6-31++G** levels of theory in the aqueous phase, respectively; Table S5: The Energies: Ground (E^GR^) and Excitation (E^EX^) state energies, and Excitation and HOMO Energies in [Ha], as well as corresponding Dipole Moments Ground, Excitation, and Transition (DM^G^, DM^EX^, D_12_) in Debays of neighbor base pair extracted from selected dimmers of *ds*-oligonucleotides, calculated at the M06-2x/6-31++G** level of theory in the aqueous phase using the DFT or TD-DFT methodology; Table S6: The vertical anion energy in [Ha] and dipole moment in [D] calculation for oligo-2Ih^(R)^ on a different level of theory in the condensed phase; only the base pair frame was taken into consideration. Additionally, the time of calculation in [h] has been shown to compare the cost; Table S7: The comparison of electronic properties of oligo-2Ih(R) in [eV], only the base pair frame was taken into consideration, and calculated at a different level of theory in the condensed phase. VIP-vertical ionisation potential, VEA-vertical electron affinity, AIP-adiabatic ionisation potential, AEA-adiabatic electron affinity, VEDE-vertical electron detachment energy, VEAE-vertical electron attachment energy, and NER-nuclear energy relaxation. ●The justification of the choice of M06-2X functional for the above calculations. ●The structure of discussed ds-oligonucleotides as suitable pdb files (cartesian coordinates) have been attached to the Supplementary Materials, as follows: (1) oligo-Ih(R)_Anion, (2) oligo-Ih(S)_Anion, (3) oligo-Ih(R)_Cation, (4) oligo-Ih(S)_Cation, (5) oligo-Ih(R)_Neutral, (6) oligo-Ih(S)_Neutral.
