# Supplementary material for: The 2Ih and OXOG Proximity Consequences on Charge Transfer through ds-DNA: Theoretical Studies of Clustered DNA Damage
Source: Molecules. 2023 Feb 26;28(5):2180. doi: 10.3390/molecules28052180 (PMC10004366; doi:10.3390/molecules28052180)
Supplement: Supplementary file 1 [file molecules-28-02180-s001.zip › Supplementary Files/Molecules 2022 Supplementary Materials corrected.pdf]

Supplementary Materials

The 2Ih and ^{OXO}G proximity consequences on charge transfer through *ds*-DNA. Theoretical studies of clustered DNA damage.

Boleslaw T. Karwowski [1*]

DNA Damage Laboratory of Food Science Department, Faculty of Pharmacy, Medical University of Lodz, ul. Muszynskiego 1, 90-151 Lodz, Poland; Boleslaw.Karwowski@umed.pl

Table 1S. Hirshfeld charge and spin distribution in the shape of *ds*-oligonucleotides, only nucleosides bases were taken into consideration, calculated at the M06-2x/6-31++G** level of theory in the aqueous phase. Vertical Cation (VC^{NC}) (NE-non-equilibrated), Vertical Cation (VC^{EQ}) (EQ-equilibrated), Vertical Anion (VA^{NE}), Vertical Anion (VA^{EQ}), Adiabatic Cation (AC), Adiabatic Anion (AA) and Vertical Neutral from Cation (VNC^{NE}), Vertical Neutral from Cation (VNC^{EQ}), Vertical Neutral from Anion (VNA^{NE}), Vertical Neutral from Anion (VNA^{EQ})

**oligo- $Ih^{(S)}$**									
	**Neutal**	**VC^{NE}**		**VC^{EQ}**		**AC**		**VNC^{NE}**	**NVC^{EQ}**
	**Charge**	**Charge**	**Spin**	**Charge**	**Spin**	**Charge**	**Spin**	**Charge**	**Charge**
**A_1T_5**	0.00	0.01	0.00	0.00	0.00	0.00	0.00	-0.01	0.00
**$Ih^{(S)}{}_2C_4$**	-0.14	-0.12	0.00	-0.13	0.00	-0.12	0.00	-0.14	-0.13
**A_3T_3**	0.11	0.16	0.02	0.16	0.02	0.15	0.02	0.10	0.10
**$^{O}G_4C_2$**	0.00	0.85	0.96	0.88	0.96	0.89	0.96	0.04	0.02
**A_5T_1**	0.03	0.10	0.02	0.09	0.02	0.08	0.02	0.00	0.01
**oligo- $Ih^{(R)}$**									
**A_1T_5**	-0.08	-0.07	0.00	-0.08	0.00	-0.09	0.00	-0.09	-0.09
**$Ih^{(R)}{}_2C_4$**	-0.07	-0.05	0.00	-0.06	0.00	-0.05	0.00	-0.07	-0.06
**A_3T_3**	0.12	0.18	0.03	0.18	0.02	0.18	0.02	0.13	0.13
**$^{O}G_4C_2$**	0.01	0.85	0.95	0.88	0.96	0.88	0.96	0.04	0.01
**A_5T_1**	0.02	0.10	0.02	0.09	0.02	0.07	0.02	0.00	0.00
**oligo- $Ih^{(S)}$**									
	**VA^{NE}**		**VA^{EQ}**		**AA**		**VNA^{NE}**	**NVA^{EQ}**	
	**Charge**	**Spin**	**Charge**	**Spin**	**Charge**	**Spin**	**Charge**	**Charge**	
**A_1T_5**	-0.08	0.03	0.00	0.00	-0.01	0.00	0.00	-0.01	
**$Ih^{(S)}{}_2C_4$**	-0.97	0.93	-0.14	0.00	-0.14	0.00	-0.12	-0.13	
**A_3T_3**	0.04	0.04	0.07	0.02	0.05	0.02	0.10	0.10	
**$^{O}G_4C_2$**	-0.01	0.00	-0.37	0.39	-0.89	0.95	-0.04	-0.02	
**A_5T_1**	0.02	0.00	-0.56	0.59	-0.01	0.03	0.07	0.06	
**oligo- $Ih^{(R)}$**									
**A_1T_5**	-0.09	0.00	-0.08	0.00	-0.08	0.00	-0.08	-0.08	
**$Ih^{(R)}{}_2C_4$**	-0.13	0.03	-0.09	0.00	-0.10	0.00	-0.09	-0.06	
**A_3T_3**	-0.52	0.71	-0.04	0.15	0.07	0.03	0.13	0.10	
**$^{O}G_4C_2$**	-0.22	0.24	-0.40	0.43	-0.87	0.94	-0.03	0.00	
**A_5T_1**	-0.04	0.03	-0.40	0.41	-0.01	0.03	0.07	0.05	

**Table 2S.** The energy barriers (in eV) for radical cation migration between base pairs within trimers. Vertical (**Vert**) mode, i.e. the energies of each base pair's radical cation, which were calculated for their neutral geometry. Adiabatic (**Adia**) mode i.e. the energies of each base pair's radical cation were calculated for their cation geometry. Arrows indicate direction of Electron-hole or Excess Electron Transfer from one base pair to another e.g., $A^+ \rightarrow G$ calculated at M06-2x/6-31++G** level of theory in the aqueous phase.									
**Electron-hole transfer**									
		$A_1 \leftarrow Ih^{(S)}_2$	$A_1 \rightarrow Ih^{(S)}_2$	$Ih^{(S)}_2 \leftarrow A_3$	$Ih^{(S)}_2 \rightarrow A_3$	$A_3 \leftarrow {}^{O}G_4$	$A_3 \rightarrow {}^{O}G_4$	${}^{O}G_4 \leftarrow A_5$	${}^{O}G_4 \rightarrow A_5$
**oligo- $Ih^{(S)}$**	Vert	-0.28	0.26	0.32	-0.30	1.45	-0.70	-0.69	1.46
	Adia	-0.28	0.28	0.32	-0.32	1.09	-1.09	-1.09	1.09
		$A_1 \leftarrow Ih^{(R)}_2$	$A_1 \rightarrow Ih^{(R)}_2$	$Ih^{(R)}_2 \leftarrow A_3$	$Ih^{(R)}_2 \rightarrow A_3$	$A_3 \leftarrow {}^{O}G_4$	$A_3 \rightarrow {}^{O}G_4$	${}^{O}G_4 \leftarrow A_5$	${}^{O}G_4 \rightarrow A_5$
**oligo- $Ih^{(R)}$**	Vert	-0.28	0.30	0.38	-0.22	1.58	-0.71	-0.67	1.44
	Adia	-0.28	0.28	0.38	-0.38	1.10	-1.10	-1.08	1.08
		$A_1 \leftarrow A_3$	$A_1 \rightarrow A_3$	$Ih^{(S)}_2 \leftarrow {}^{O}G_4$	$Ih^{(S)}_2 \rightarrow {}^{O}G_4$	$A_3 \leftarrow A_5$	$A_3 \rightarrow A_5$		
**oligo- $Ih^{(S)}$**	Vert	0.05	0.00	1.40	-1.02	0.03	0.03		
	Adia	0.05	-0.05	1.41	-1.41	0.00	0.00		
		$A_1 \leftarrow A_3$	$A_1 \rightarrow A_3$	$Ih^{(R)}_2 \leftarrow {}^{O}G_4$	$Ih^{(R)}_2 \rightarrow {}^{O}G_4$	$A_3 \leftarrow A_5$	$A_3 \rightarrow A_5$		
**oligo- $Ih^{(R)}$**	Vert	0.09	-0.01	1.49	-1.07	0.17	0.15		
	Adia	0.10	-0.10	1.48	-1.48	0.02	-0.02		
**Excess electron transfer**									
		$A_1 \leftarrow Ih^{(S)}_2$	$A_1 \rightarrow Ih^{(S)}_2$	$Ih^{(S)}_2 \leftarrow A_3$	$Ih^{(S)}_2 \rightarrow A_3$	$A_3 \leftarrow {}^{O}G_4$	$A_3 \rightarrow G_4$	${}^{O}G_4 \leftarrow A_5$	${}^{O}G_4 \rightarrow A_5$
**oligo- $Ih^{(S)}$**	Vert	-0.02	0.03	0.01	0.01	1.02	-0.10	-0.05	0.95
	Adia	0.01	-0.01	-0.03	0.03	0.55	-0.55	-0.52	0.52
		$A_1 \leftarrow Ih^{(S)}_2$	$A_1 \rightarrow Ih^{(R)}_2$	$Ih^{(R)}_2 \leftarrow A_3$	$Ih^{(R)}_2 \rightarrow A_3$	$A_3 \leftarrow {}^{O}G_4$	$A_3 \rightarrow {}^{O}G_4$	${}^{O}G_4 \leftarrow A_5$	${}^{O}G_4 \rightarrow A_5$
**oligo- $Ih^{(R)}$**	Vert	-0.13	0.12	0.10	-0.10	1.00	-0.08	-0.06	0.96
	Adia	-0.12	0.12	0.10	-0.10	0.53	-0.53	-0.53	0.53
		$A_1 \leftarrow A_3$	$A_1 \rightarrow A_3$	$Ih^{(S)}_2 \leftarrow {}^{O}G_4$	$Ih^{(S)}_2 \rightarrow {}^{O}G_4$	$A_3 \leftarrow A_5$	$A_3 \rightarrow A_5$		
**oligo- $Ih^{(S)}$**	Vert	-0.02	-0.01	0.62	-0.19	0.01	0.01		
	Adia	-0.02	0.02	0.63	-0.63	0.0	0.00		
		$A_1 \leftarrow A_3$	$A_1 \rightarrow A_3$	$Ih^{(R)}_2 \leftarrow {}^{O}G_4$	$Ih^{(R)}_2 \rightarrow {}^{O}G_4$	$A_3 \leftarrow A_5$	$A_3 \rightarrow A_5$		
**oligo- $Ih^{(R)}$**	Vert	-0.03	-0.01	0.56	-0.08	0.04	0.00		
	Adia	0.02	0.02	0.53	-0.53	0.04	-0.04		

Table 3S. The energies (in Hartree) of Neural, Vertical Cation, Adiabatic Cation and Vertical Neutral forms of base pairs extracted from *ds*-oligonucleotides calculated at the M06-2x/6-31++G** level of theory in the aqueous phase.

**oligo- $Ih^{(S)}$**	**Neutral**	**Vertical Cation**	**Adiabatic Cation**	**Vert Neutral**
**A_1T_5**	-921.191958	-920.946932	-920.94701	-921.191915
**$Ih^{(S)}_2C_4$**	-1088.838514	-1088.583903	-1088.583366	-1088.838571
**A_3T_3**	-921.191786	-920.947498	-920.94856	-921.192173
**$^{O}G_4C_2$**	-1012.478318	-1012.260573	-1012.275011	-1012.466119
**A_5T_1**	-921.192606	-920.947853	-920.949264	-921.192507
**oligo- $Ih^{(R)}$**		**Vertical Anion**	**Adiabatic Anion**	**Vert Neutral**
**A_1T_5**		-921.24427	-921.244044	-921.191897
**$Ih^{(S)}_2C_4$**		-1088.886416	-1088.886312	-1088.838884
**A_3T_3**		-921.242829	-921.243212	-921.191569
**$^{O}G_4C_2$**		-1012.533017	-1012.549125	-1012.461181
**A_5T_1**		-921.245288	-921.244088	-921.19169
**oligo- $Ih^{(S)}$**	**Neutral**	**Vertical Cation**	**Adiabatic Cation**	**Vert Neutral**
**A_1T_5**	-921.190367	-920.943165	-920.942748	-921.190015
**$Ih^{(R)}_2C_4$**	-1088.841744	-1088.583442	-1088.583908	-1088.841452
**A_3T_3**	-921.189926	-920.94049	-920.94603	-921.190424
**G_4C_2**	-1012.478211	-1012.259808	-1012.274591	-1012.466047
**A_5T_1**	-921.19276	-920.948418	-920.949579	-921.192663
**oligo- $Ih^{(R)}$**	**Neutral**	**Vertical Anion**	**Adiabatic Anion**	**Vert Neutral**
**A_1T_5**		-921.242029	-921.241702	-921.190087
**$Ih^{(R)}_2C_4$**		-1088.892079	-1088.893298	-1088.842495
**A_3T_3**		-921.240461	-921.240432	-921.189578
**$^{O}G_4C_2$**		-1012.532613	-1012.549088	-1012.461211
**A_5T_1**		-921.245708	-921.244631	-921.191986

Table 4S. The energies (in Hartree) of Neural, Vertical Cation (VC^{NC}) (NE-non-equilibrated), Vertical Cation (VC^{EQ}) (EQ-equilibrated), Vertical Anion (VA^{NE}), Vertical Anion (VA^{EQ}), Adiabatic Cation (AC), Adiabatic Anion (AA) and Vertical Neutral from Cation (VNC^{NE}), Vertical Neutral from Cation (VNC^{EQ}), Vertical Neutral from Anion (VNA^{NE}), Vertical Neutral from Anion (VNA^{EQ}) of complete DNA double helix and base pairs skeleton extracted from *ds*-oligonucleotides calculated at the M06-2x/6-31+G** and M06-2x/6-31++G** level of theory in the aqueous phase, respectively.

Complete DNA double helix										
**Neutrał**	**VC^{NE}**	**VC^{EQ}**	**VA^{NE}**	**VA^{EQ}**	**AC**	**AA**	**VNC^{NE}**	**VNC^{EQ}**	**VNA^{NE}**	**VNA^{EQ}**
**oligo- $Ih^{(S)}$**										
-13003,137323	-13002,892945	-13002,917287	-13003,176491	-13003,188743	-13002,934711	-13003,213998	-13003,099031	-13003,123349	-13003,091910	-13003,117857
**oligo- $Ih^{(R)}$**										
-12850,347306	-12850,108293	-12850,130889	-12850,383811	-12850,396229	-12850,146309	-12850,424651	-12850,307312	-12850,330186	-12850,301506	-12850,326060
**Base Pairs skeleton extracted from *ds*-oligonucleotides**										
**oligo- $Ih^{(S)}$**										
-4864.985711	-4864.745911	-4864.768789	-4865.009606	-4865.035966	-4864.783718	-4865.053929	-4864.963346	-4864.970625	-4864.960928	-4864.966728
**oligo- $Ih^{(R)}$**										
-4864.977775	-4864.736912	-4864.759502	-4865.006612	-4865.026047	-4864.772993	-4865.045393	-4864.952344	-4864.959923	-4864.951723	-4864.958557

Table 5S. The Energies: Ground (E^{GR}) and Excitation (E^{EX}) state energies and Excitation and HOMO Energies in [Ha] as well as corresponding Dipole Moments Ground, Excitation, and Transition (DM^{G}, DM^{EX}, D_{12}) in Debays of neighbor base pair extracted from selected dimmers of *ds*-oligonucleotides, calculated at the M06-2x/6-31++G** level of theory in the aqueous phase using the DFT or TD-DFT methodology.;

SYSTEM	B.P. Dimer	E^{GR}	DM^{GR}	E^{EX}	DM^{EX}	D_{12}	E^{HOMO}	E^{HOMO-1}	E^{LUMO}	E^{LUMO-1}
**oligo-$2Ih^{(S)}$**	**A_1||$^{(S)}2Ih_2$**	-2010.054925	16.51	-2009.922881	16.82	11.93	-0.2835	-0.2971	-0.0265	-0.0203
	**$2Ih^{(S)}{}_2$||A_3**	-2010.046527	15.44	-2009.914249	15.42	4.21	-0.2622	-0.2792	-0.0154	-0.0125
	**A_3||$^{o}G_4$**	-1933.693914	15.80	-1933.566104	14.04	4.60	-0.2549	-0.2811	-0.0150	-0.0120
	**$^{o}G_4$||A_5**	-1933.695898	16.15	-1933.568302	14.48	5.11	-0.2544	-0.2819	-0.0177	-0.0130
**oligo-$2Ih^{(R)}$**	**A_1||$^{(R)}2Ih_2$**	-2010.043753	14.66	-2009.910346	15.02	6.35	-0.2844	-0.2920	-0.0144	-0.0116
	**$2Ih^{(R)}$||A_3**	-2008.604783	15.51	-2008.656942	15.35	2.24	-0.2895	-0.2974	-0.1845	-0.0183
	**A_3||$^{o}G_4$**	-1933.693907	14.91	-1933.565957	13.11	5.29	-0.2558	-0.2869	-0.0156	-0.0119
	**$^{o}G_4$||A_5**	-1933.696017	16.16	-1933.567807	14.48	2.80	-0.2552	-0.2814	-0.0172	-0.0128

---XXXXXXXX---

The justification of M06-2X functional choice for above calculation.

Due to the fact that the charge transfer process through the double helix occurs by the π-π interaction between base pairs, the anions – vertical or adiabatic – should show the valence character.
It is noteworthy that in his studies, Voityuk showed that the diffuse function present (6-31++G** basis set) dramatically changes the character of the anion from a valence to dipole-bond when the unrestricted Hartree-Fock method was used [1].
On the other hand, it is commonly accepted that diffuse functions are highly demanded, for a proper anion state description by extended basis sets. Because of the above and the postulate that the charge during migration is dispersed over three base pairs for these studies the following DFT functionals were tested: wB97-XD, M-11, M06-L, and M06-2X[2]. Additionally, two types of basis sets were used: Pople tripel-ζ 6-31 and Dunning double-ζ D95 with polarization and diffuse functions. The spin density was calculated for a vertical anion as the most problematic and time-consuming calculation and the neutral ground state of oligo-2Ih$^{(R)}$ was optimized at the M06-2x/D95** level of theory in the condensed phase. As shown in **Figure 1S** only in the case of the calculation performed at the wB97-XD/6-31++G** level of theory in the aqueous phase did the vertical anion show the dipole-bond character, which is in good agreement with Voityuk's previous data. For all other cases, the anion valence type was observed irrespective of the used functional or diffuse function implemented to the basis set. However, only in the case at M-11/6-31+G** and M-11/6-31++G** levels was the unpaired electron strictly located at the single base T_4, while in other cases it was dispersed over the three base-pairs as expected. It can be concluded that the decreases in HF exchange in short-range (SR) with 100% in long-range (LG) in the range-separated hybrid functionals leads to a different ds-DNA vertical anion character than is commonly accepted. The hybrid generalized gradient approximation (GGA) range-separated hybrid functional ωB97-XD contained 22% of Hartree-Fock exchange SR and 100% LR, while semi-empirical meta-GGA M-11 contained 42.8% SR and 100% LR. In contrast, global hybrid meta-GGA functionals such as M06-2x and M06-L contained only 54% and 0% of HF exchange fraction respectively [3]. Furthermore, the cost-intensive calculation of oligo-2Ih$^{(R)}$ vertical anion energy (time consumption) was found in the following order (time given in hours): M-11/6-31++G**(151) > M-11/6-31++G* (132) ~ M06-2X/D95++** (132) > wB97-XD/6-31+G** (107) > wB97-XD/6-31++G** (89) > M06-2X/D95+** (86) > M06-2X/6-31++G** (66) > M06-2X/6-31+G** (52) > M06-L/6-31++G** (31) > M06-L/6-31+G** (21) > M06-2X/D95** (18) as shown in **Table 6S**. It should be pointed out that for this test only the base-pairs skeleton was used (for the complete structure it takes 1638 hours per M06-2X/6-31+G** level). Because of the large number of calculations, the M06-2X functional with moderated basis set (6-31++G**) in the aqueous phase using the CPCM continuum solvation model was chosen as the most effective. For a full double helix structure, the 6-31+G** basis set was chosen as a limit of calculation power possibility. Moreover, the further discussed adiabatic electronic properties in [eV] of oligo-2Ih$^{(R)}$: ionization potential (5.57) and electron affinity (-1.84) achieved at the above level of theory were comparable with those obtained with an extended aug-cc-pVDZ [4] basis set, i.e. 5.91 and -1.84 [eV] respectively please see **Table 7S**.

1 A. A. Voityuk, *J. Chem. Phys.*, , DOI:10.1063/1.1961400.

2 A. Kumar, A. Adhikary, M. D. Sevilla and D. M. Close, *Phys. Chem. Chem. Phys.*, 2020, **22**, 5078–5089.

3 N. Mardirossian and M. Head-Gordon, *Mol. Phys.*, 2017, **115**, 2315–2372.

4 T. H. Dunning, *J. Chem. Phys.*, 1989, **90**, 1007–1023.

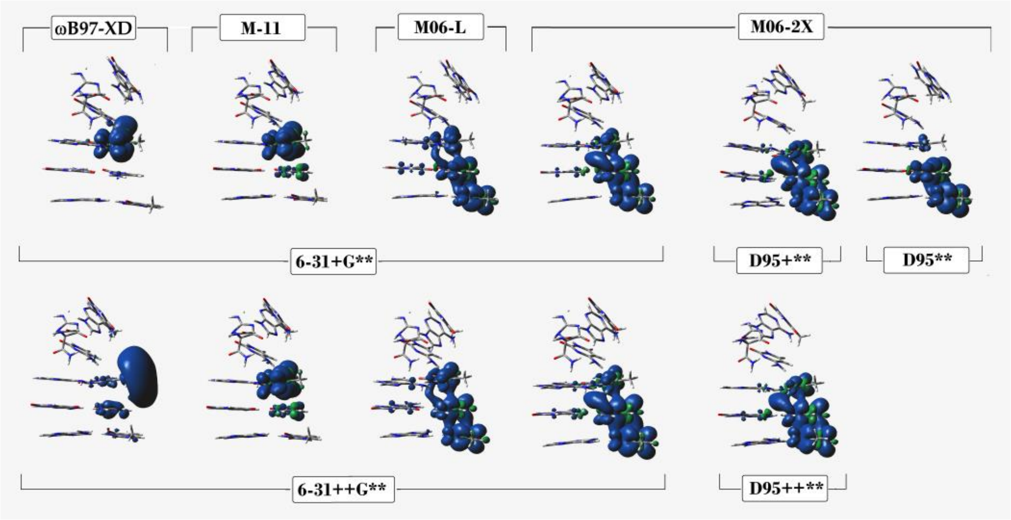

Figure 1SM. Graphical visualization of oligo-2Ih$^{(R)}$(vertical anion) spin distribution calculated at different levels of theory in the aqueous phase. The initial neutral state of oligo-2Ih$^{(R)}$ geometry was obtained at M06-2x/D95** level of theory in the condensed phase.

Table 6S. The vertical anion energy in [Ha] and dipole moment in [D] calculation of oligo-2Ih$^{(R)}$ on different level of theory in condensed phase, only the base-pair frame was taken into consideration. Additionally, the and time of calculation in [h] have been all shown to compare the cost.

Functional	oligo-2Ih$^{(R)}$ (Vertical Anion)				
	**parameter**	**Basis set**			
		**aug-cc-pVDZ**	**6-31++G****	**6-31+G****	**D95****
**M06-2x**	**Energy [Ha]**	-4865.671568	-4865.026047	-4865.023826	-4865.750421
	**DM [D]**	17.86	19.59	19.46/21.20 [a)]	23.68
	**Time of Calculation[h]**	327	66	52/1638 [a)]	18
					[b)] **D95+****
**M06-L**	**Energy [Ha]**	-4865.115110	-4866.445567	-4866.443779	-4865.865317
	**DM [D]**	15.77	17.63	17.67	19.97
	**Time [h]**	294	31	24	86
					[b)] **D95++****
**M-11**	**Energy [Ha]**	-4865.102351	-4864.642931	-4864.640910	-4865.868772
	**DM [D]**	9.91	10.46	10.50	20.13
	**Time [h]**	686	151	132	132
**ωB97-XD**	**Energy [Ha]**	-4865.800318	-4865.385164	-4865.415755	
	**DM [D]**	19.21	15.77	9.46	
	**Time [h]**	464	89	107	

[a)] the complete pentamer was taken into consideration; [b)] the M06-2x functional was used

Table 7S. The comparsion of electronic properties of oligo-2Ih(R) in [eV], only the base-pair frame was taken into consideration, calculated at different level of theory in condensed phase. VIP-vertical ionisation potential, VEA-vertical electron affinity, AIP-adiabatic ionisation potential, AEA-adiabatic electron affinity, VEDE-vertical electron detachment energy, VEAE-vertical electron attachment energy, NER-nuclear energy relaxation.

Electronic properties of oligo-2Ih$^{(R)}$ in [eV]										
**Level of Theory**	**VIP**	**VEA**	**AIP**	**AEA**	**NER1**	**NER2**	**NER3**	**NER4**	**VEAE**	**VEDE**
**M062x / 6-31+G****	-5.94	-1.31	-5.57	-1.84	-0.37	-0.48	-0.53	-0.53	5.09	2.36
**M062x / 6-31++G****	-5.94	-1.31	-5.57	-1.84	-0.37	-0.49	-0.53	-0.52	5.09	2.36
**M062x / aug-cc-pVDZ**	-5.88	-1.31	-5.51	-1.84	-0.37	-0.48	-0.53	-0.52	5.03	2.35
**M062x / D95****	-5.88	-1.18	-5.51	-1.78	-0.37	-0.50	-0.60	-0.48	5.00	2.26
**M-11 / 6-31++G****	-6.10	-1.55	-5.68	-1.90	-0.41	-0.47	-0.36	-0.55	5.22	2.46
**M06-L / 6-31++G****	-5.43	-1.63	-5.20	-1.75	-0.22	-0.47	-0.12	-0.40	4.73	2.15
**ωB97-XD / 6-31++G****	-5.80	-0.46	-5.45	-1.83	-0.36	-0.49	-1.37	-0.44	4.96	2.27
